# Awakening Australia to Rare Diseases: Symposium report and preliminary outcomes

**DOI:** 10.1186/1750-1172-6-57

**Published:** 2011-08-18

**Authors:** Hugh JS Dawkins, Caron M Molster, Leanne M Youngs, Peter C O'Leary

**Affiliations:** 1 Office of Population Health Genomics, Western Australian Department of Health, PO Box 8172, Stirling Street, Perth, WA, Australia; 2 Centre for Population Health Research, Curtin Health Innovation Health Research Institute, Curtin University, Perth, WA, Australia; 3 School of Pathology and Laboratory Medicine, University of Western Australia, WA, Australia; 4 School of Women's and Infants' Health, University of Western Australia, WA, Australia

## Background

Western Australia recently hosted Australia's inaugural national rare diseases symposium, entitled ***Awakening Australia to Rare Diseases: Global perspectives on establishing a coordinated approach to a national plan ***http://www.raredisease.com.au in the port city of Fremantle, Western Australia from the 17th-20th of April 2011.

The Office of Population Health Genomics (OPHG), Department of Health Western Australia, in conjunction with the Australian Paediatric Surveillance Unit (APSU), initiated the Symposium and established National and Local Organizing Committees to compose a program that included plenary sessions of invited speakers followed by small group discussions to explore issues raised in the formal presentations. The sessions and content were informed by the Western Australian supporting organisation branches of Carers Australia, Cystic Fibrosis Australia, Motor Neurone Disease Australia, Multiple Sclerosis Society of Western Australia, Muscular Dystrophy WA and Senses Foundation with clinical and disability insights from Genetic Services of Western Australia and the Disability Services Commission. This national rare disease symposium built on the work begun by the Human Genetics Society of Australasia (HGSA) on rare genetic disorders and more recently by the ASPU led Rare Diseases Working Group [1].

International speakers and advisors presented their experiences and perspectives as representatives of a range of organisations and institutions including the U.S. National Organisation for Rare Disorders (NORD), the International Conferences on Rare Diseases (ICORD), New Zealand Organisation for Rare Diseases (NZORD), European Organisation on Rare Diseases (EURORDIS), Orphanet and the U.S. National Institutes of Health Office of Rare Diseases Research. More than 160 speakers and delegates attended from a range of countries including Australia, North America, United Kingdom, New Zealand, France, Hong Kong and Singapore. The majority of delegates were people living with a rare disease who generously shared their personal narratives and contributed invaluable information on many of the issues that will lead to the formulation of a National Plan.

### Key Outcomes

The Symposium registration session included the launch of "Rare Friends", a non-partisan network of State politicians, formed to raise awareness of the impact of rare disorders in the community and to support solutions that improve life outcomes for those living with rare disorders http://www.raredisease.com.au. In addition, Rare Friends are committed to extending their network through State and Federal parliamentary colleagues.

Dr Noel Nannup, a respected indigenous Noongar Elder and scholar, provided a stirring welcome to country in the Noongar language and a translation and explanation of the ceremony with reference to the gathering of delegates at the Symposium. Mr Kim Snowball, Director General of the Department of Health in Western Australia then launched the Symposium by highlighting the importance of rare diseases within the context of health service delivery [2,3]. He spoke of the impact of chronic, often life-shortening and generally debilitating diseases and the frequent associations with other co-morbidities such as developmental delays, depression and intellectual disability [4-6]. According to best estimates rare diseases cumulatively affect between 6-8% of the Australian population [1,4,7]. Mr Snowball observed that the issues confronting those with rare diseases are universal and are best researched and addressed through global collaborations. He announced that this Symposium was the starting point for a Western Australian rare disease plan that will feed into a national rare disease strategy [8-12]. Mr Snowball committed WA Health to working with health professionals, service providers and governments to improve the health and well being of people with rare diseases.

Over the three days of the Symposium program the delegates had the opportunity to listen to the vast experiences of the international speakers and, more importantly, people living with rare diseases. (Refer to full program http://www.raredisease.com.au). These talks covered a wide range of policy issues including participant and professional perspectives of the history of a national plan in Australia, the role of umbrella organisations (NORD: The National Organization for Rare Disorders in the United States of America and EURORDIS: Rare Diseases Europe), the roles of general practitioners, specialist physicians and disability services, clinical trials and registries, and the effective application of coordinated networks to advocate on behalf of those with rare diseases. Several individuals shared their personal experiences of diagnosis, therapeutic interventions and prognoses. Among the most poignant was the story of a young man with a progressive neuromuscular disorder, who began by asking the delegates not to focus on his genetic diagnosis as he was one of many who lived with some form of rare disease. His final message, "I don't need to be cured, I just want to live" encapsulated the need to accommodate multidimensional themes in a rare disease strategy.

Collectively the plenary talks were crucial in framing the stakeholder engagement workshop sessions which were conducted as deliberative-style discussions [13]. During these sessions, participants discussed their perspectives on the goals of a national plan, empowerment and advocacy, medical and social support agencies, research and translation, networks and partnerships that led to a consensus on recommending the development of a national plan for rare diseases. At the end of each workshop session each group prepared a written summary of their deliberations and outcomes which were collated for review.

Preliminary analyses of participant responses to an end-of-forum survey indicate satisfaction that the engagement process was transparent, respectful and very useful to identify the key issues for the rare diseases community. Participants also reflected that it was important to establish a cooperation network that created a single voice on behalf of those with rare diseases in Australia.

Although the feedback is still being analysed, there was consensus on key issues:

■ An enthusiastic endorsement to develop a National Plan informed by the symposium workshops and ongoing engagement processes, building on existing international frameworks and national plans such as the EUROPLAN and to facilitate international collaborations;

■ The agreement to form a single overarching advocacy group for rare diseases in Australia that ensures a voice for all persons living with a rare disease;

■ A recognition that advocacy to government on behalf of all rare diseases requires collaboration with the pharmaceutical industry and academic researchers to drive developments;

■ Agreement on the need for improvements in the collection and analyses of the incidence and prevalence of rare diseases, the costs to families, community, national productivity, and the health and disability services through voluntary national rare disease registries;

■ Specific attention was directed towards exploring how service delivery could be improved through integrated, multi-disciplinary clinical and support networks with shared care, management and services requirements and support for the proposed National Disability Insurance Scheme http://www.everyaustraliancounts.com.au.

## Conclusion

The meeting concluded with an open session and a discussion of delegates' views about the actions that would advance the development of the National Plan. The Director of OPHG, Dr Peter O'Leary summarised the successful outcomes of the deliberative engagement sessions, noting the five themes (above) that will be carried forward with the enthusiastic endorsement of all the delegates.

As a farewell message, we were reminded that Dr Nannup had provided each delegate with the gift of a traditional Indigenous message stick to commemorate the gathering, the discussions and the shared decisions to go forward. These sticks serve as symbols of the communal responsibility to maintain the spirit of collaboration, communication and goodwill shared at the Symposium.

## Competing interests

The authors declare that they have no competing interests.
